# Contact allergy to methylisothiazolinone with three clinical presentations in one patient

**DOI:** 10.1111/cod.13384

**Published:** 2019-08-27

**Authors:** Amélie Gorris, Julia Valencak, Verena Schremser, Christine Bangert, Tamar Kinaciyan

**Affiliations:** ^1^ Department of Dermatology Medical University of Vienna Vienna Austria

**Keywords:** airborne contact dermatitis, allergic contact dermatitis, connubial allergic contact dermatitis, methylisothiazolinone, patch test

1

Methylisothiazolinone (MI) is a frequent cause of allergic contact dermatitis (ACD). It is a well‐known contact allergen in hygienic and cosmetic products but less known in other settings. Here, we present a patient who suffered primarily from occupational ACD and years later from dermatitis due to exposure to MI in private life.

## CASE REPORT

**Figure 1:**
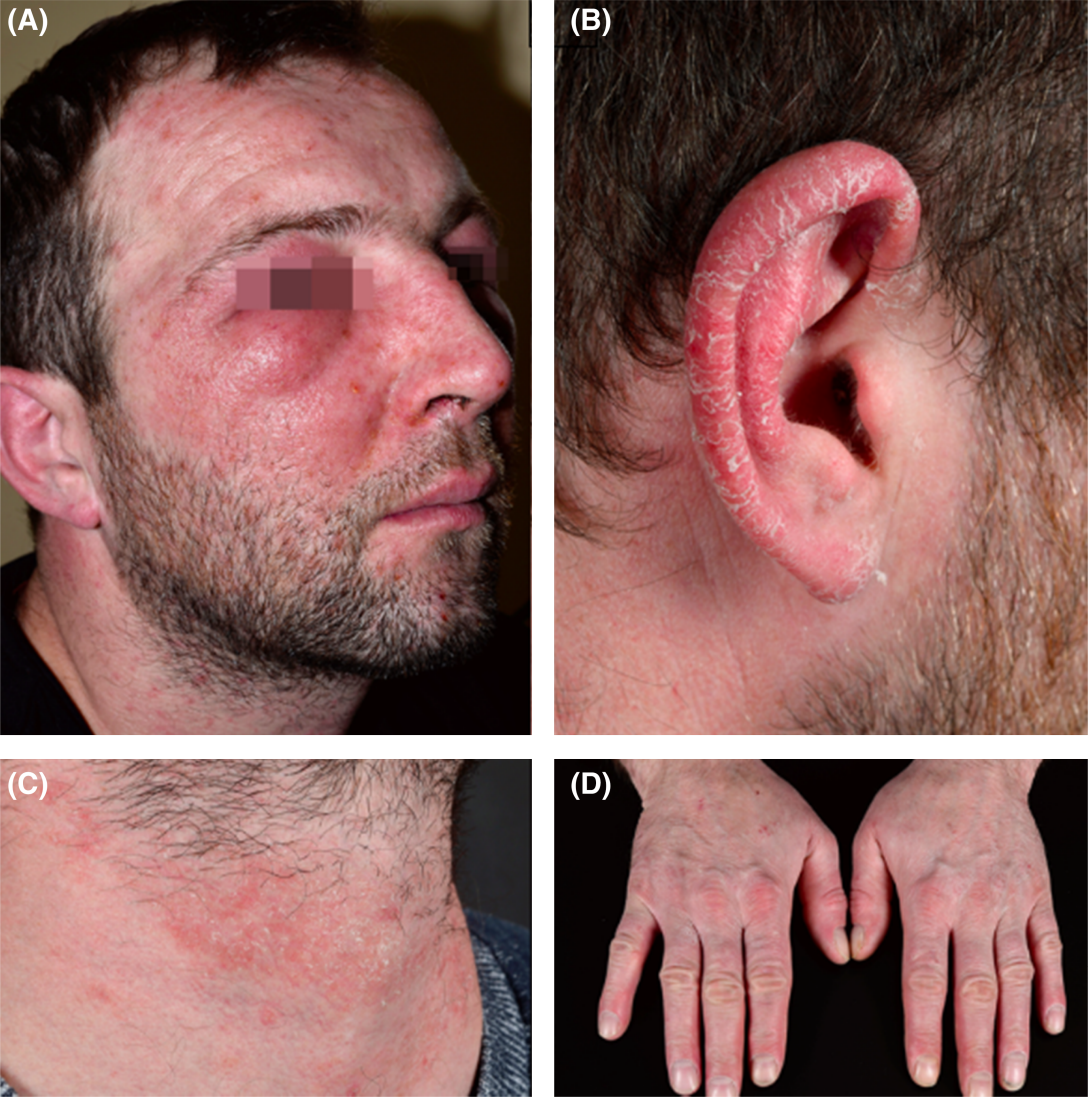
(**A‐D**), Allergic contact dermatitis caused by airborne exposure to methylisothiazolinone from freshly painted walls

A 34‐year‐old man presented to our allergy outpatient clinic with massive dermatitis of his face, ears, neck, and hands associated with collateral eyelid oedema (Figure 1A‐D). He reported similar reactions when he previously worked as a construction worker and once after his girlfriend coloured her hair.

Initially, an irritant dermatitis on atopic background was suspected, but the patient had suffered from atopic dermatitis only during childhood. Unsatisfied by the lack of efficacy of topical steroids and moisturizers, he was referred to our department.

Patch tests were performed at two different sessions according to ESCD guidelines.^1^ Finn Chambers on Scanpor tape (SmartPractice, Phoenix, Arizona) and Fixomull were used. First, the European baseline series, a plastics/glues and a hairdresser's series were tested. Because of a doubtful reaction, *p*‐aminophenol was repeated together with the European baseline series, a cutting fluids, and a biocides series. The patient only reacted positively to MI 0.05% and octylisothiazolinone 0.025% at day (D) 2 and D3. MI is known to cross‐react with octylisothiazolinone.^2^

A re‐evaluation of the case history revealed that 10 years previously, the patient developed repeated episodes of eczema on hands and face while he was working as a construction worker and was in contact with cement, and adhesives and wall paints. He had been working as an iron binder for the past 6 years. During this time, he only once had mild eczema on the face after contact with the freshly coloured hair of his girlfriend. After talking to the girlfriend, we obtained the name of her hair color (Belle Color of Garnier) in which MI was an ingredient.

Five days before referral to our department, the patient moved to an apartment that had recently been repainted. The next morning, he woke up with itching and redness of the face, neck, and ears that worsened rapidly. After contacting the owner of the apartment, we obtained the name of the wall paint (“Alpinaweiß das Original”), which again contained MI.

As topical steroids did not lead to sustained healing of the dermatitis, we recommended treatment of the apartment walls with inorganic sulfur salts as reported by Schwensen et al.^3^ However, the patient preferred to move again, and since then remained free of symptoms.

## DISCUSSION

Our case highlights (a) the importance and need to raise awareness of the presence of MI in water‐based wall paints; (b) persistence of allergic sensitization for more than 10 years; and (c) the fact that—depending on the allergen source—ACD, connubial ACD, or airborne contact dermatitis may arise from MI in the same patient, necessitating different treatment/management strategies.

MI is a commonly used preservative that is effective against bacteria, molds, and yeast growth. It was introduced in the early 1980s, primarily in industrial products such as water‐cooling systems, cutting oils, glues, latex emulsions, and papermills.^4^ From 2009 to 2011, sensitizations to MI in the preservative series increased from 1.9% to 4.4%, due to its wide use in cosmetics, body care products, hair dyes, and even in wall paints and the textile industry.^5^, ^6^

Because MI was assumed to be less sensitizing than chlorinated additives, the EU allowed its use up to a maximum of 100 ppm in cosmetic products. Nevertheless, an increasing number of sensitized patients were reported^7^ and according to a recent IVDK report, it is still among the most common contact allergens.^5^

Since early 2000, reports have been published about ACD, also airborne, arising from evaporation of MI after use of water‐based wall paints commonly containing MI as preservatives. Unfortunately, MI is still widely used in paints in relatively high concentrations across Europe, without any warning on the paints.^8^

The treatment of airborne ACD arising from MI evaporation from wall paints can be challenging due to continuous exposure. One solution is the treatment of the painted walls with inorganic sulfur salt, which leads to inactivation of the allergenic properties of MI.^3^ Alternatively, the use of a cream containing 2% of the antioxidant glutathione, which is capable of destroying the N‐S bond and neutralizing the allergenic structure of MI, has been reported to be effective in the treatment of airborne ACD.^9^

Based on the case history, the patch test results, and verification of MI in the products to which the patient was exposed, we conclude that he was primarily sensitized to MI and developed ACD while working as a construction worker and painting house walls. This was followed by an episode of connubial ACD^10^ after skin contact with his girlfriend's freshly colored hair, and finally he developed airborne ACD after moving to an apartment that had been recently repainted.

## References

[1] Johansen JD , Aalto‐Korte K , Agner T , et al. European Society of Contact Dermatitis guideline for diagnostic patch testing ‐ recommendations on best practice. Contact Dermatitis. 2015;73:195‐221. 26179009 10.1111/cod.12432

[2] Goossens A , Apers S , Lambert J , et al. Octylisothiazolinone, an additional cause of allergic contact dermatitis caused by leather: case series and potential implications for the study of cross‐reactivity with methylisothiazolinone. Contact Dermatitis. 2016;75:276‐284. 27538353 10.1111/cod.12670

[3] Schwensen JF , Menné T , Andersen KE , Sommerlund MJJD . Occupations at risk of developing contact allergy to isothiazolinonesin in Danish contact dermatitis patients. Contact Dermatitis. 2014;71:295‐302. 25141913 10.1111/cod.12286

[4] Aerts O , Goossens A , Lambert J , Lepoittevin JP . Contact allergy caused by isothiazolinone derivatives: an overview of non‐cosmetic and unusual cosmetic sources. Eur J Dermatol. 2017;27:115‐122. 28174143 10.1684/ejd.2016.2951

[5] Geier J , Schubert S , Lessmann H , et al. Die häufigsten kontaktallergene der Jahre 2015 – 2017: Daten des informationsverbundes dermatologischer kliniken. Dermatol Beruf Umwelt. 2019;67:3‐11.

[6] Urwin R , Warburton K , Carder M , Turner S , Agius R , Wilkinson SM . Methylchloroisothiazolinone and methylisothiazolinone contact allergy: an occupational perspective. Contact Dermatitis. 2015;72:381‐386. 25810132 10.1111/cod.12379

[7] Aerts O , Goossens A , Giordano‐Labadie F . Contact allergy caused by methylisothiazolinone: the Belgian‐French experience. Eur J Dermatol. 2015;25:228‐233. 26412037 10.1684/ejd.2015.2608

[8] Schwensen JF , Lundov MD , Bossi R , et al. Methylisothiazolinone and benzisothiazolinone are widely used in paint: a multicentre study of paints from five European countries. Contact Dermatitis. 2015;72:127‐138. 25510184 10.1111/cod.12322

[9] Isaksson M . Successful inhibition of allergic contact dermatitis caused by methylchloroisothiazolinone/methylisothiazolinone with topical glutathione. Contact Dermatitis. 2015;73:126‐128. 25891419 10.1111/cod.12405

[10] Ferreira C , Rezende I , Guilherme A , Rosmaninho I , Lopes I . Allergic contact connubial dermatitis caused by hair products. Contact Dermatitis. 2019;80:186‐187. 30548282 10.1111/cod.13163

